# A survey into the influence of dopaminergic drug exposure on ‘sense of presence’ symptoms in patients with parkinson's disease

**DOI:** 10.1192/bjo.2021.744

**Published:** 2021-06-18

**Authors:** Emma Padfield, Hannah Potticary, Tim Segal

**Affiliations:** Kings College London, South London and Maudsley NHS Foundation Trust

## Abstract

**Aims:**

The first objective was to estimate prevalence of sense of presence (SoP) experiences in patients with Parkinson's Disease (PD), including whether onset was prior to or after commencing dopaminergic medication. The second objective was to explore the relationship between frequency of SoP experiences and dopaminergic drug, drug dosage and length of drug exposure. The experimental hypothesis was that SoP symptoms in PD would present more frequently in patients treated longer and with higher dopaminergic drug doses.

**Background:**

PD is a debilitating neurodegenerative disorder. Psychiatric symptoms are common and associated with impaired quality of life and higher treatment costs. PD psychosis often starts with ‘minor hallucinations’, the most common being a false ‘sense of presence’ (SoP), the vivid sensation that someone else is nearby when nobody is there. SoP symptoms typically do not cause significant distress but may act as a prognostic marker for future severe psychosis and may prompt alteration of treatment or reduction in dopaminergic drug dosage. This study aimed to extend prior research by characterizing SoP further and investigating the link with dopaminergic medication.

**Method:**

This was a retrospective, cross-sectional study. Twenty-one patients diagnosed with PD completed a questionnaire to identify presence of SoP symptoms, duration of symptoms, timing of onset related to dopaminergic treatment and the frequency of symptoms in relation to current levodopa equivalent dose (LED). Descriptive frequencies were compared using a two-tailed t-test. Multiple regression analysis was conducted to assess the relationship between frequency of SP experiences, levodopa equivalent dose and length of drug exposure.

**Result:**

Sixteen of twenty-one patients reported experiencing SoP symptoms. Patients who had not experienced SoP symptoms had a significantly lower LED than those who had experienced these symptoms. There were no other significant differences between the groups. No statistical significance was shown on regression analysis; however our study was not adequately powered for the regression analysis as the number of participants was too low.

**Conclusion:**

This study confirms that SoP symptoms are common among patients with PD and supports a correlation between the total daily equivalent dose of levodopa and SoP symptoms. It does not provide evidence for a temporal relationship between onset of SoP symptoms and duration of dopaminergic treatment. The study was insufficiently powered and a larger study is required to investigate further.

